# A new fossil species of the extant genus *Vicelva* from mid‐Cretaceous Kachin amber (Coleoptera: Staphylinidae)

**DOI:** 10.1002/ece3.11487

**Published:** 2024-06-25

**Authors:** Yan‐Da Li, Alfred F. Newton, Di‐Ying Huang, Chen‐Yang Cai

**Affiliations:** ^1^ State Key Laboratory of Palaeobiology and Stratigraphy, Nanjing Institute of Geology and Palaeontology Chinese Academy of Sciences Nanjing China; ^2^ Bristol Palaeobiology Group, School of Earth Sciences University of Bristol Bristol UK; ^3^ Negaunee Integrative Research Center Field Museum of Natural History Chicago Illinois USA

**Keywords:** beetle, coprolite, Cretaceous, Kachin amber, mineralization, Phloeocharinae

## Abstract

A new species of the extant staphylinid genus *Vicelva* Moore & Legner, *V. rasilis* sp. nov., is reported from mid‐Cretaceous Kachin amber of northern Myanmar. *Vicelva rasilis* is distinguishable from extant members of *Vicelva* by the smoother dorsal surface of head, pronotum and elytra, less prominent median projection of clypeus, unnotched mesal edge of mandibles, semiglabrous antennomere 6, and longer tarsomere 1. The pollen‐containing coprolite attached to the beetle and the crystals within the beetle body provide valuable information about the biology and taphonomy of the fossil.

## Graphical abstract

A new species of the extant staphylinid genus *Vicelva* Moore & Legner, *V. rasilis* sp. nov., is reported from mid‐Cretaceous Kachin amber of northern Myanmar. The pollen‐containing coprolite attached to the beetle and the crystals within the beetle body provide valuable information about the biology and taphonomy of the fossil.

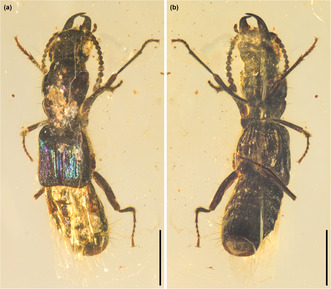

## 1 INTRODUCTION

The family Staphylinidae, commonly known as rove beetles, stands out as an extraordinary example of diversification in the history of life. Currently, 34 extant subfamilies are recognized in Staphylinidae (Newton, 2022). Among them, the subfamily Phloeocharinae has historically served as a taxonomic receptacle for relatively primitive rove beetles difficult to be classified elsewhere (Herman, 1972; Newton et al., 2000; Thayer, 2016). Over time, several genera have been gradually removed from Phloeocharinae and reassigned to subfamilies including Omaliinae (*Giulianium* Moore), Olisthaerinae (*Olisthaerus* Dejean), Tachyporinae (*Derops* Sharp), Oxytelinae (*Euphanias* Fairmaire & Laboulbène) and Pseudopsinae (*Pseudopsis* Newman) (e.g., Herman, 1972, 1975; Moore, 1964; Newton, 1982; Newton & Thayer, 1995; Smetana, 1983). Presently, only seven extant genera are left in this subfamily: *Charhyphus* Sharp, *Dytoscotes* Smetana & Campbell, *Ecbletus* Sharp, *Phloeocharis* Mannerheim, *Phloeognathus* Steel, *Pseudophloeocharis* Steel, and *Vicelva* Moore & Legner (Newton, 2022; Newton et al., 2000). However, the Phloeocharinae as currently defined is still unlikely to be monophyletic, as demonstrated by both morphological (Ashe, 2005; Ashe & Newton Jr, 1993) and molecular (Lü et al., 2020; McKenna et al., 2015) studies, even though only *Phloeocharis* and *Charhyphus* were sampled in these analyses.

**FIGURE 1:**
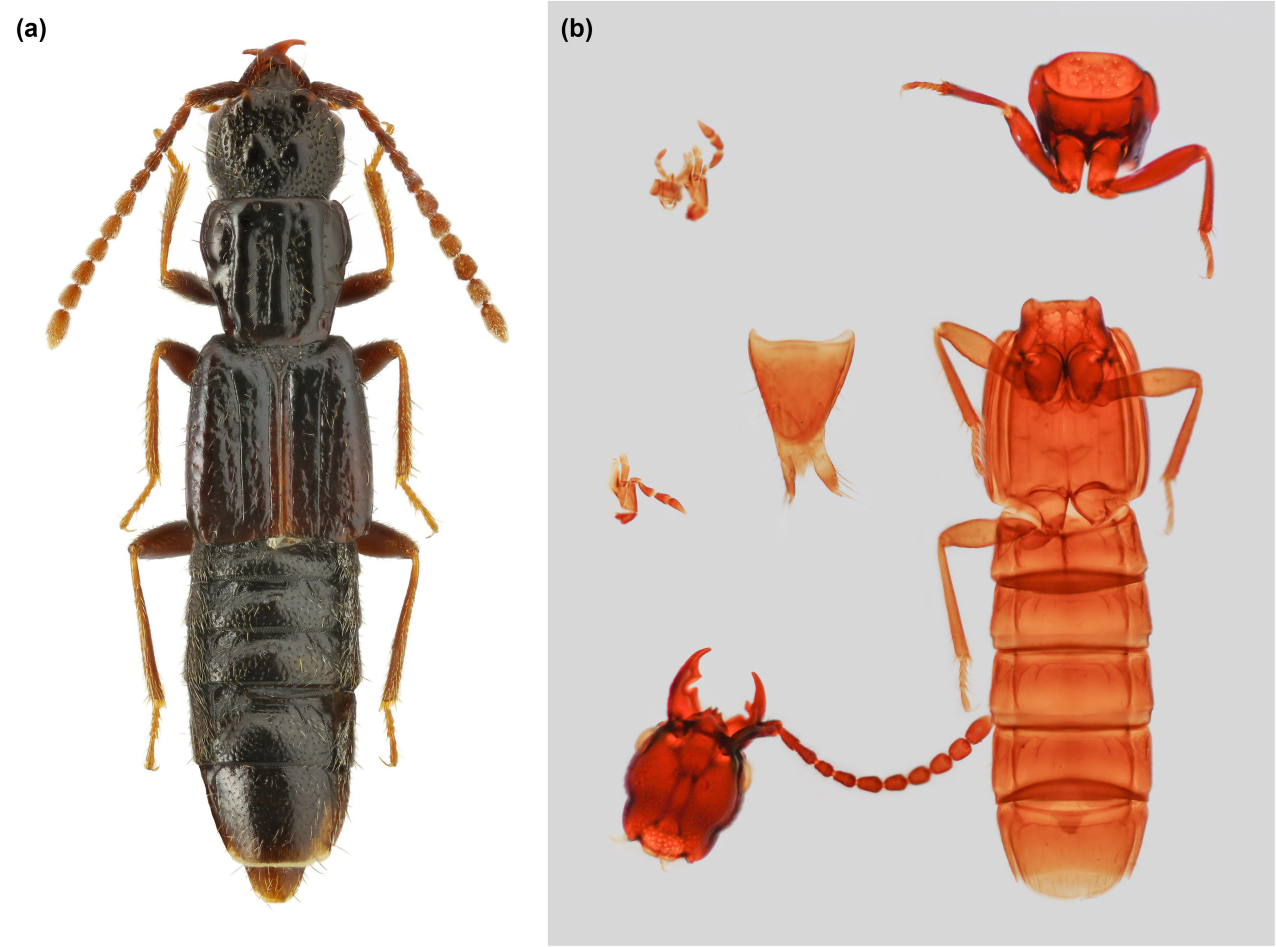
Extant members of *Vicelva*. (a) *Vicelva altaica*, holotype. Photography by Kirill V. Makarov; (b) Dissection of *Vicelva vandykei*, female, collected from Alder Creek, Oregon.

Within Phloeocharinae, *Vicelva* represents a morphologically isolated genus that has not been well studied (Figure 1). There are only two described species of *Vicelva* in the extant fauna: the type species *V. vandykei* (Hatch) in western North America and probably the Far East of Russia (Herman, 2001; Ryabukhin, 1999), and *V. altaica* (Kastcheev) in Kazakhstan (Kastcheev, 2003). The type species was originally placed in Oxytelinae as *Coprophilus vandykei* Hatch (Hatch, 1957), and was later recognized as conspecific to *V. paradoxica* Moore & Legner (Moore, 1974; Moore & Legner, 1973). The second species was placed in a monospecific genus also in Oxytelinae by the original author (Kastcheev, 2003), and was transferred to *Vicelva* by Newton (2015).

Among Phloeocharinae, members of the extant genus *Charhyphus* have been described from Eocene Baltic amber (Shavrin & Kairišs, 2020; Yamamoto et al., 2022), and members of the extant genus *Phloeocharis* have been described from Late Cretaceous New Jersey amber (Chatzimanolis et al., 2013) and mid‐Cretaceous Kachin amber (Yamamoto & Newton, 2023). An extinct genus, *Angucharcotes* Li et al. was also described from Kachin amber (Li et al., 2023). Cai et al. (2020) mentioned a specimen of *Vicelva* from Kachin amber. In this study, we provide detailed figures and a formal description for this specimen. The taphonomic implications of this specimen are also discussed.

## 2 MATERIALS AND METHODS

**FIGURE 2:**
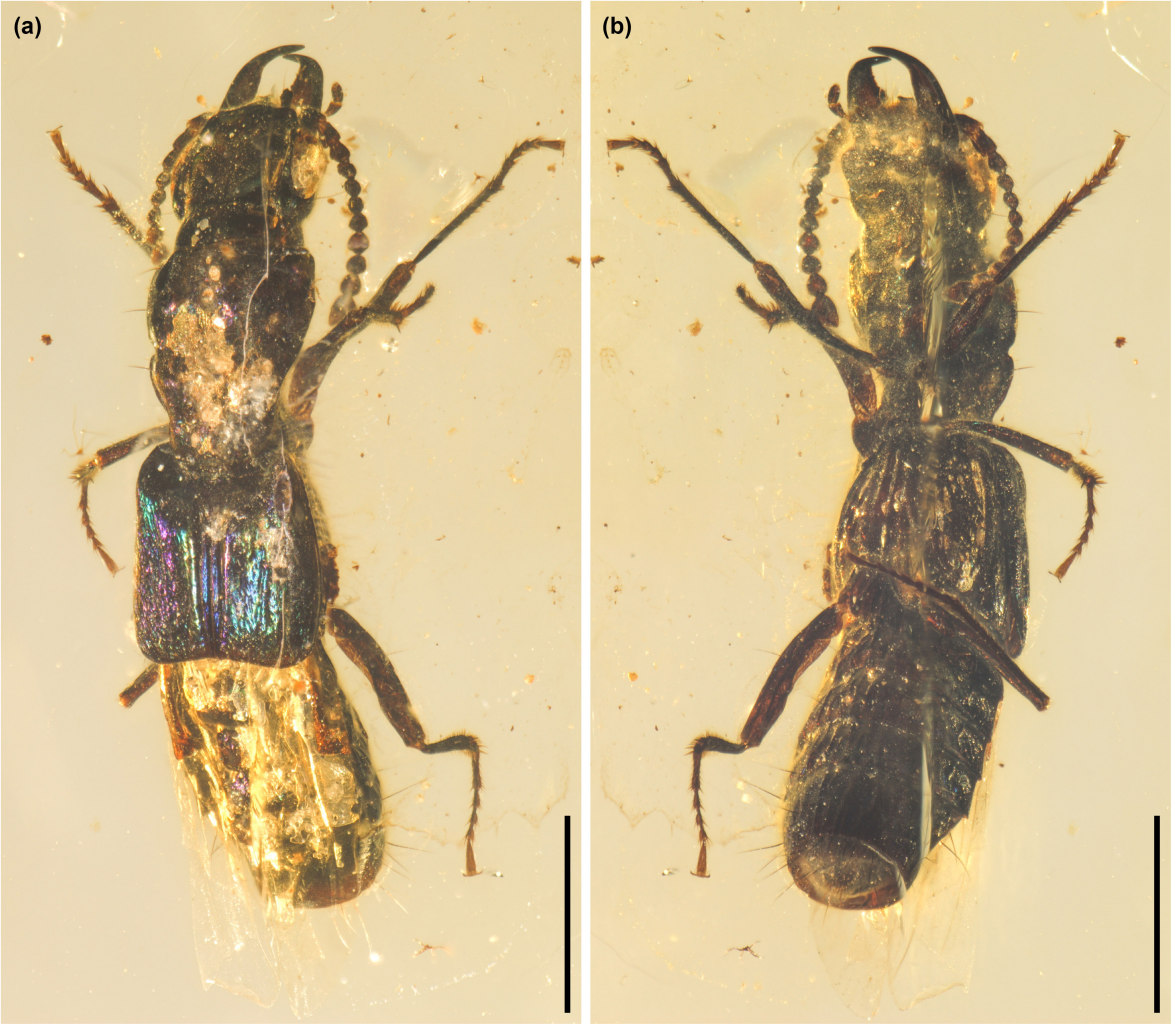
General habitus of *Vicelva rasilis* **sp. nov.**, holotype, NIGP166145, under incident light. (a) Dorsal view; (b) Ventral view. Scale bars: 1 mm.

**FIGURE 3:**
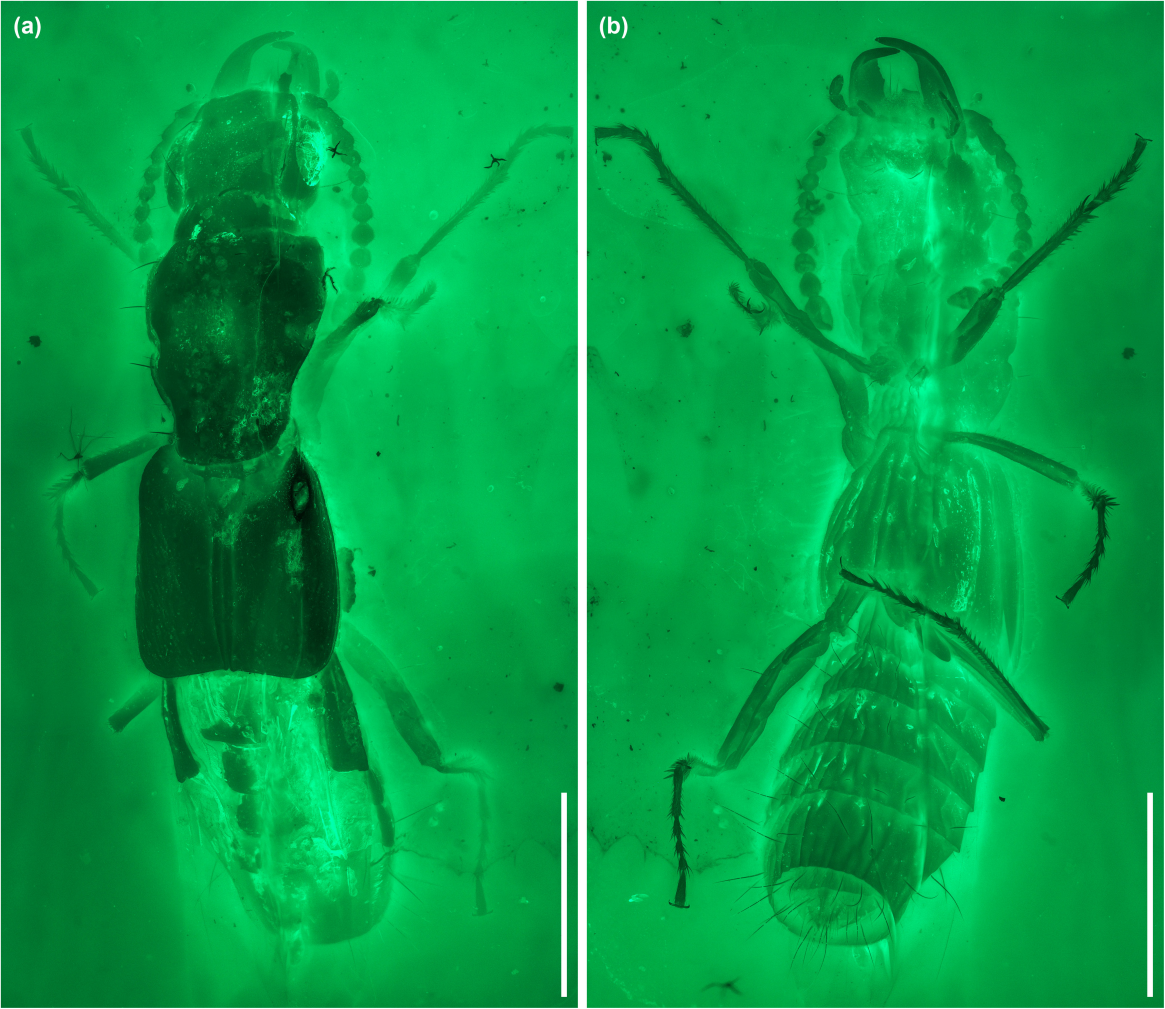
General habitus of *Vicelva rasilis* **sp. nov.**, holotype, NIGP166145, under widefield fluorescence. (a) Dorsal view; (b) Ventral view. Scale bars: 1 mm.

**FIGURE 4:**
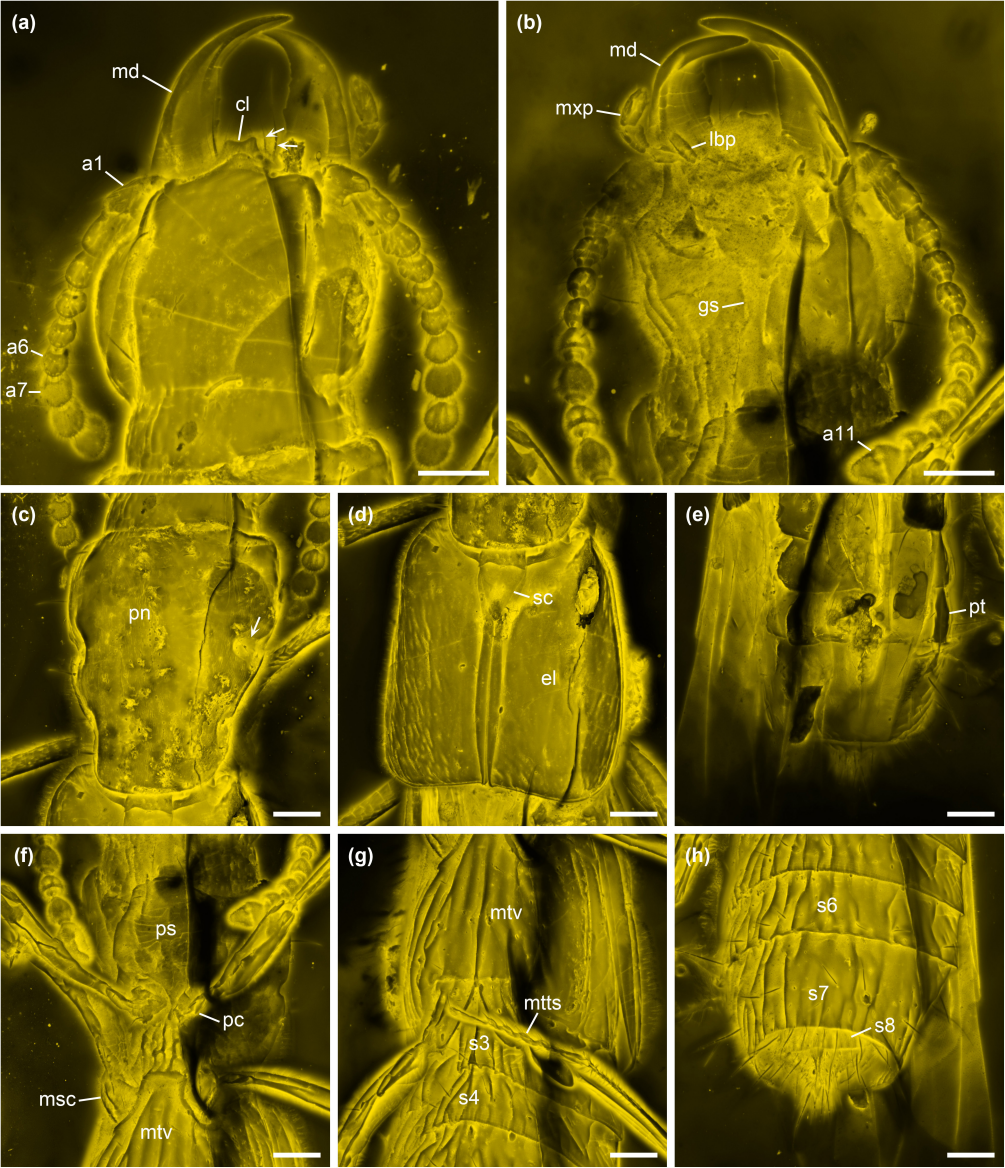
Details of *Vicelva rasilis* **sp. nov.**, holotype, NIGP166145, under confocal microscopy. (a) Head, dorsal view, with the macrosetae of labrum labeled by the arrows; (b) Head, ventral view; (c) Prothorax, dorsal view, with the lateral pronotal depression labeled by the arrow; (d) Elytra, dorsal view; (e) Abdomen, dorsal view; (f) Pro‐ and mesothorax, ventral view; (g) Metathorax and abdominal base, ventral view; (h) Abdominal apex, ventral view. Abbreviations: a1–11, antennomeres 1–11; cl, clypeus; el, elytron; gs, gular suture; lbp, labial palp; md, mandible; msc, mesocoxa; mtv, metaventrite; mtts, metatarsus; mxp, maxillary palp; pc, procoxa; pn, pronotum; ps, prosternum; pt, paratergite; s3–8, abdominal sternites III–VIII; sc, scutellum. Scale bars: 200 μm.

**FIGURE 5:**
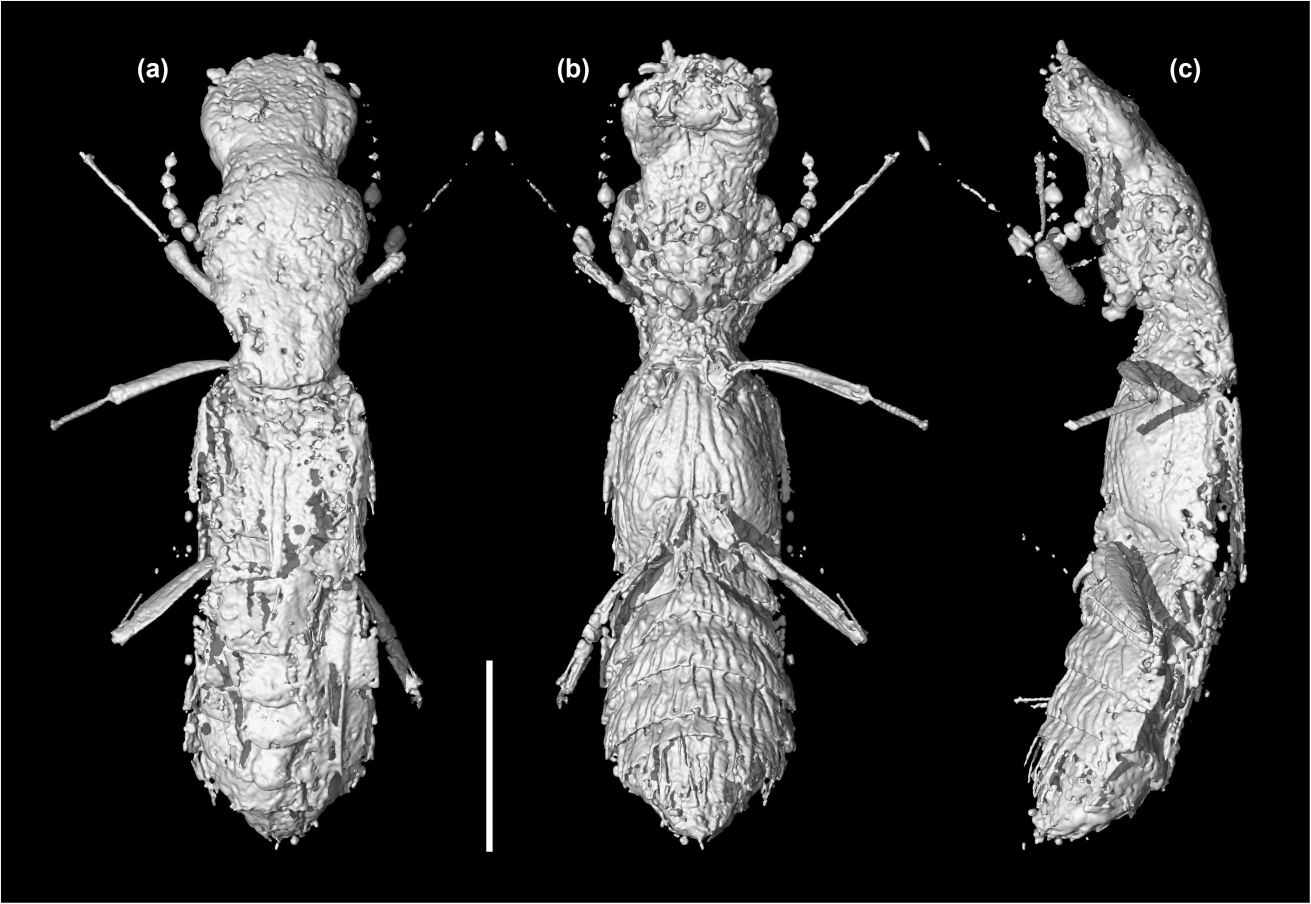
X‐ray microtomographic reconstruction of *Vicelva rasilis* **sp. nov.**, holotype, NIGP166145. (a) Dorsal view; (b) Ventral view; (c) Lateral view. Scale bar: 1 mm.

**FIGURE 6:**
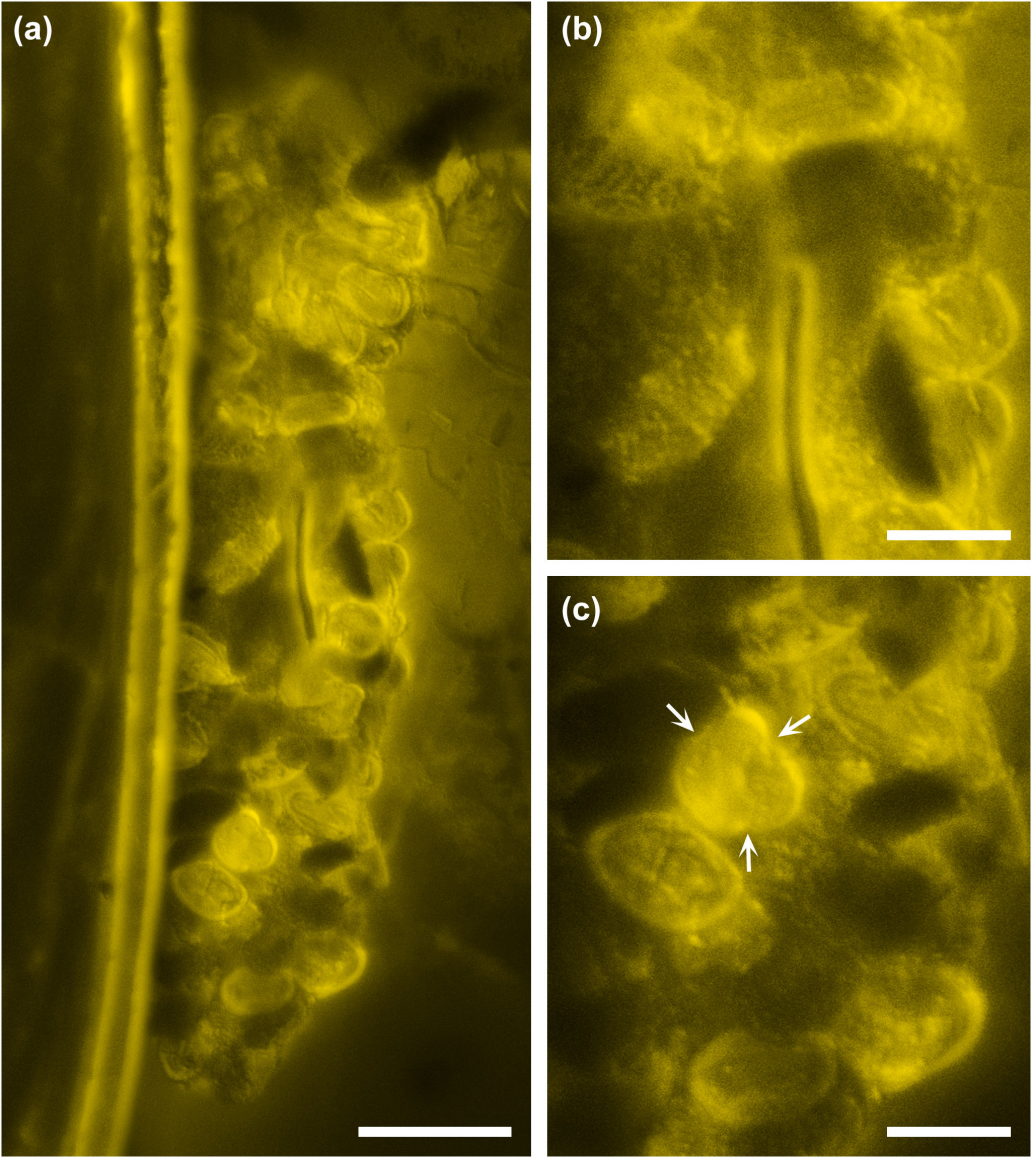
Pollen‐containing coprolite associated with *Vicelva rasilis*, NIGP166145. The arrows in (c) indicate the three colpi in polar view. Scale bars: 50 μm in (a); 20 μm in (b, c).

**FIGURE 7:**
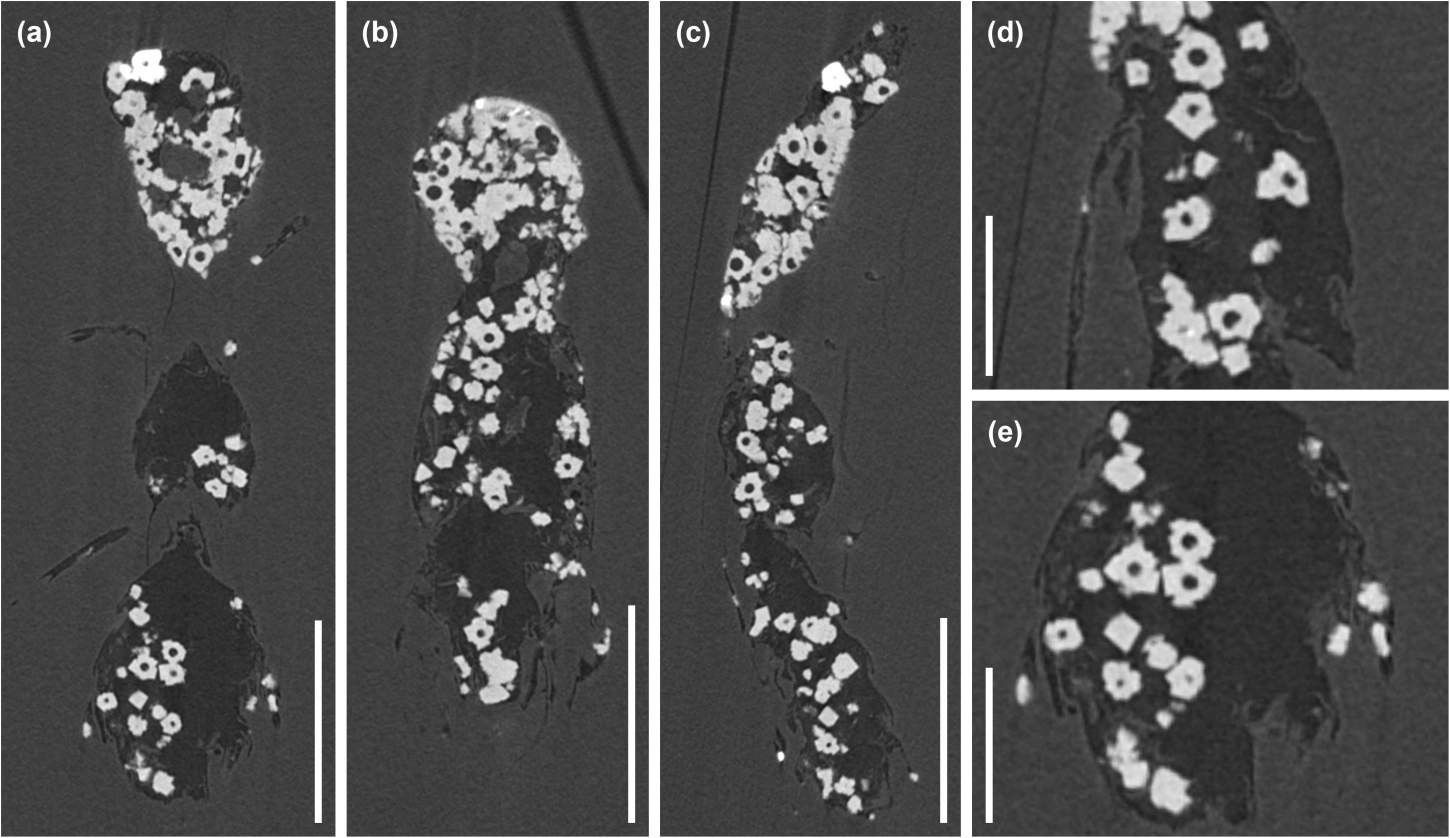
Virtual slices of *Vicelva rasilis*, NIGP166145, based on X‐ray microtomographic reconstruction, showing the crystals within the beetle body. (a, b, e) Horizontal sections; (c, d) Sagittal sections. Scale bars: 1 mm in (a–c); 400 μm in (d, e).

The Kachin amber (Burmese amber) specimen studied herein (Figures 2, 3, 4, 5, 6, 7) originated from amber mines near Noije Bum (26°20′ N, 96°36′ E), Hukawng Valley, Kachin State, northern Myanmar. The amber specimen is deposited in the Nanjing Institute of Geology and Palaeontology (NIGP), Chinese Academy of Sciences (CAS), Nanjing, China. The amber piece was trimmed with a small table saw, ground with emery paper of different grit sizes, and finally polished with polishing powder.

Photographs under incident light were taken with a Zeiss Discovery V20 stereo microscope. Widefield fluorescence images were captured with a Zeiss Axio Imager 2 light microscope combined with a fluorescence imaging system. Confocal images were obtained with a Zeiss LSM710 confocal laser scanning microscope, using the 488 nm (Argon) or 561 nm (DPSS 561‐10) laser excitation line (Fu et al., 2021). Images were stacked with Helicon Focus 7.0.2, Zerene Stacker 1.04 and Adobe Photoshop CC, and were further processed in Adobe Photoshop CC to adjust brightness and contrast. Microtomographic data were obtained with a Zeiss Xradia 520 Versa 3D X‐ray microscope at the micro‐CT laboratory of NIGP and analyzed in VGStudio MAX 3.0. Scanning parameters were as follows: isotropic voxel size, 6.8202 μm; power, 4 W; acceleration voltage, 50 kV; exposure time, 1.5 s; projections, 2401.

## 3 SYSTEMATIC PALEONTOLOGY

Order Coleoptera Linnaeus, 1758

Superfamily Staphylinoidea Latreille, 1802

Family Staphylinidae Latreille, 1802

Subfamily Phloeocharinae Erichson, 1839

Genus *Vicelva* Moore & Legner, 1973

***Vicelva rasilis* sp. nov.**

Figures 2, 3, 4, 5.

**Material.** Holotype, NIGP166145.

**Etymology.** The specific name, formed from the Latin adjective *rasilis*, refers to the overall simple and smooth dorsal surface of the head and prothorax.

**Locality and horizon.** Amber mine located near Noije Bum Village, Tanai Township, Myitkyina District, Kachin State, Myanmar; unnamed horizon, mid‐Cretaceous, Upper Albian to Lower Cenomanian.

**Diagnosis.** Head dorsally smooth, without swellings (Figure 4a). Antennomeres 1–6 semiglabrous; antennomeres 7–11 densely pubescent (Figure 4a,b). Median projection of clypeus much less prominent, with lower pair of teeth more distinctly separated from it (Figure 4a). Mandibles with mesal edge relatively smooth, at most with small denticles (Figure 4a,b). Pronotum and elytra without distinct longitudinal elevations and grooves (Figure 4c,d). Meso‐metaventral junction externally as straight line (Figure 4f). Tarsomere 1 relatively long, subequal in length to tarsomere 5 (Figures 3b and 4g).

**FIGURE 8:**
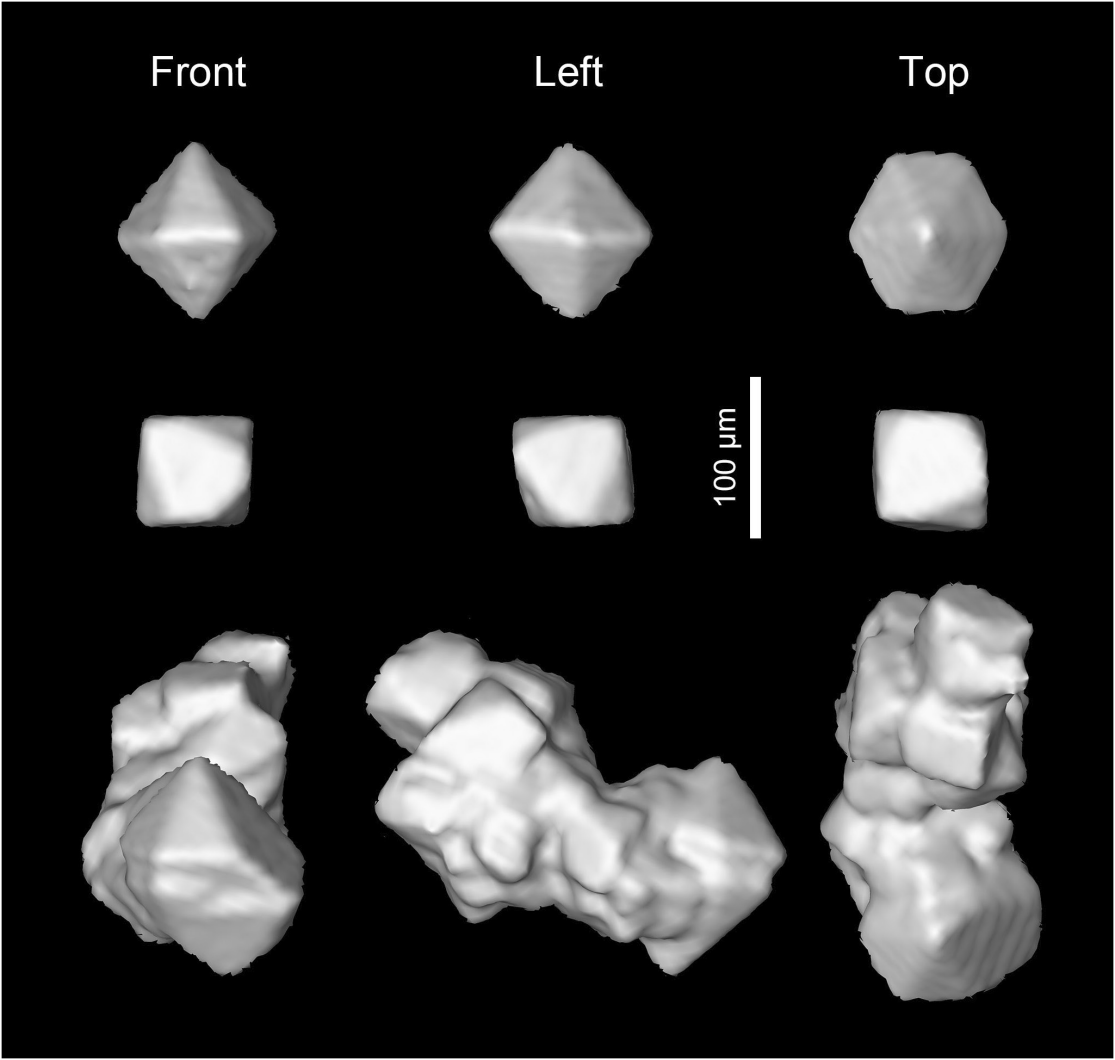
Examples of crystals within the body of *Vicelva rasilis*, NIGP166145, based on X‐ray microtomographic reconstruction.

**Description.** Body elongate, about 4.5 mm long, 1.0 mm wide. Dorsal surface of head, prothorax and elytra glabrous except for a few stout setae.

Head prognathous, elongate, with weak neck constriction; dorsal surface simple, smooth, without protuberances. Clypeus with weak median projection at same level as rest of head and bilobed projection (lateral teeth) immediately below that. Labrum largely concealed dorsally, except for anterolateral macrosetae projecting beside lower clypeal projection. Eyes moderate, only slightly protruding, without interfacetal setae. Antennal insertions located anterolaterally; subantennal grooves absent. Antennae 11‐segmented; antennomeres 1–2 elongate; antennomeres 3–10 submoniliform; antennomere 11 conical; antennomeres 1–6 semiglabrous, with sparse long setae only; antennomeres 7–11 densely pubescent. Mandibles strongly projecting, with acute apices; mesal edge relatively smooth, at most with small denticles. Apical maxillary and labial palpomeres cylindrical to conical, not aciculate. Gular sutures narrowly separated, weakly diverging anteriad.

Pronotal disc longer than wide, widest anteriorly, gradually narrowing posteriad; lateral margins smooth; anterior and posterior angles rounded, not produced; surface overall smooth, without longitudinal ridges or grooves, but with paired distinct depressions adjacent to lateral margins. Prosternum anteriorly slightly produced. Procoxal cavities very narrowly separated.

Scutellar shield triangular, with subbasal ridge, smooth otherwise. Elytra subparallel, short, reaching abdominal segment III; margins smooth; inner and anterior margins bordered; lateral epipleural keel probably present. Hind wings fully developed (unfolded and covering most of abdomen). Mesocoxal cavities separated by about coxal diameter. Meso‐metaventral junction externally as straight line. Metacoxae slightly contiguous.

Legs relatively long, slender. Tibial spurs well developed and paired. Tarsi 5‐5‐5; tarsomeres simple; tarsomeres 1 and 5 elongate, subequal in length; tarsomeres 2–4 each about half as long as tarsomere 1 or 5. Pretarsal claws simple, equal.

Abdomen with six segments (III–VIII) exposed before genital segments, but tergites largely concealed by unfolded wings; segments contracted and segment VIII largely invaginated, with details about tergal ridges, parasclerites, and abdominal intersegmental membranes not visible. Apices of sternite VIII and genital segments (IX–X) exposed but details unclear.

## 4 DISCUSSION

### 4.1 Systematic position of *Vicelva rasilis* and comparison with extant relatives

The staphylinid subfamily Phloeocharinae is notoriously difficult to characterize as a group, and even in the modern more restricted concept the subfamily is probably not monophyletic. For example, Herman (1972) when proposing this modern definition cited only two possible derived characters shared by the genera (the structure of the hypopharynx, and the presence of certain cuticular processes on some abdominal terga) in support of the group, and both of these characters are invisible in the fossil described here due to its preservation with the inner mouthparts concealed and unfolded wings covering the abdominal terga. Nevertheless, the individual extant and extinct genera placed in this subfamily (see Introduction) are generally distinctive in appearance and characters, and this is especially true for the genus *Vicelva*. The two extant species of this genus are unique in appearance among staphylinids (e.g., for *V. altaica* see Figure 1a, and for *V. vandykei* see photos available online provided by the California Academy of Sciences, 2023a, 2023b). *Vicelva rasilis* shares this appearance (Figures 2 and 3) and the following unique combination of characters with extant *Vicelva*: body very elongate and parallel‐sided; head large, elongate, with long slender antennae, broad weakly defined neck, strongly and acutely produced mandibles, protruding and multidentate clypeus of unique shape, and more or less concealed labrum with only anterolateral macrosetae visible from above (Figure 4a); pronotum elongate, widest in anterior half (Figure 4c); elytra short, reaching abdominal tergite III (Figure 4d); and legs slender with 5‐segmented elongate tarsi (Figures 3b and 4g). The two extant species of *Vicelva* also have two pairs of basolateral ridges on the basal abdominal terga, two pairs of laterosclerites on most abdominal segments, and abdominal intersegmental membranes with a brick‐wall pattern, but these characters in the fossil are not available due to the preservation state. The fossil has various wrinkles or crenulations or other surface irregularities, especially on the thoracic and abdominal venter (Figures 2b and 4f–h), that are not present in the extant species, but which we interpret as taphonomic artifacts of preservation. Overall, the numerous and often unique characters shared by the extinct and extant species give us confidence that *V. rasilis* belongs in the genus *Vicelva*.

Both extant species of *Vicelva* are characterized by a strongly produced clypeus with a median tooth (prominent projection) and 2–3 further teeth immediately below that, concealing most of the labrum; vertex of head with a pair of smooth round swellings; and pronotum and elytra with longitudinal elevations and grooves (Figure 1; Kastcheev, 2003; Moore & Legner, 1973). In contrast, in *V. rasilis*, the clypeus is similar (in our interpretation) but the median projection is much less prominent and the lower pair of teeth are more distinctly separated from the upper median projection (Figure 4a), the head lacks the paired swellings (Figure 4a), and the pronotum lacks the longitudinal elevations and grooves (Figure 4c). The longitudinal ridges or grooves on the elytra, even if present in *V. rasilis* (which might be a taphonomic artifact), are much more indistinct (Figures 2a and 4d). The mesal edge of mandibles has a deep notch in *V. vandykei* and *V. altaica* (Figure 1b; Kastcheev, 2003: figure 3), which is absent in *V. rasilis* (Figure 4a,b). In *V. vandykei* and *V. altaica*, the first five antennomeres are semiglabrous, with sparse long setae only, and the apical six are densely pubescent (Figure 1a). In *V. rasilis*, the first six antennomeres are semiglabrous, and the apical five are densely pubescent (Figure 4a,b). *Vicelva rasilis* differs additionally from the extant congeners in the much longer tarsomere 1, which is subequal in length to tarsomere 5 (Figures 3b and 4g), while in the extant species, the tarsomere 1 is longer than 2–4 but only about half as long as tarsomere 5. Finally, the mesocoxae are well separated and the meso‐metaventral junction externally appears to be a straight line in *V. rasilis* (Figure 3f), but the mesocoxae are only narrowly separated and the mesoventral process is sharply acute in *V. vandykei* (Figure 1b) and possibly also *V. altaica* according to the drawing by Kastcheev (2003).

### 4.2 Pollen‐containing coprolite associated with *Vicelva rasilis*

A cylindrical coprolite is attached to the lateral side of the metathorax of NIGP166145, composed of tricolpate pollen which likely belong to the eudicots (Figure 6). The general morphology of the coprolite is similar to fecal pellets of extant and fossil beetles reported previously (Klavins et al., 2005; Procheş & Johnson, 2009; Tihelka et al., 2021). The pollen grains are somewhat deformed, representing possible signs of digestion (e.g., Human & Nicolson, 2003; Johnson & Nicolson, 2001).

However, this coprolite is unlikely to be produced by *Vicelva rasilis*. Although nothing is known about the biology of extant *Vicelva*, the large and sharp mandibles of *V. rasilis* and its extant relatives suggest that they are suited to predation of other insects. It looks possible that *V. rasilis* searches on plants for prey, potentially including some pollen‐feeding beetles.

### 4.3 Crystals in the fossil and taphonomic implications

Insects trapped in amber generally have their external morphology well‐preserved. However, the preservation quality of their internal anatomy varies greatly (McCoy et al., 2016, 2018). Some of them preserve exquisite soft tissue (e.g., Grimaldi et al., 1994; Li et al., 2021; Poinar & Hess, 1982; Van de Kamp et al., 2014), and some others preserve the more or less sclerotized genitalia (e.g., Bukejs et al., 2020; Li et al., 2022; Nabozhenko et al., 2020; Schmidt et al., 2019, 2021; Yin et al., 2022), while in the remaining cases, the interior may be a cavity or (partially) filled with high‐Z materials, with no original structure discernible. The interior of the present specimen, NIGP166145, is mainly a hollow cavity, with more or less scattered crystals within it (Figure 7). The crystals are denser in the prothorax, and sparser in the metathorax and abdomen.

Very few studies paid attention to the mineralization happened in amber inclusions (e.g., Baroni‐Urbani & Graeser, 1987; Garty et al., 1982; Martínez‐Delclòs et al., 2004). According to a recent study (Jiang et al., 2022), calcification and silicification are common in the preservation of fossils in the Kachin amber, forming mainly calcite, chalcedony and quartz. Some minor amount of carbonaceous material, pyrite, iron oxide and phyllosilicate minerals may also be present. In NIGP166145, two well‐isolated crystals appear to be a hexagonal bipyramid or a pseudocube (Figure 8); therefore we suppose that these crystals are most likely quartz (Goldschmidt, 1922). Some organic matter might have served as the crystallization centers, which were then decayed after the formation of quartz, leaving an empty sphere within the crystal (Figure 7).

The specimen NIGP166145 has metallic greenish‐blue color on the elytra, resulting from exceptional preservation of epicuticular multilayer reflectors (Cai et al., 2020). Cai et al. (2020) implied that the insects in amber with well‐preserved structural color (and therefore well‐preserved external cuticle) would likely also have internal organs preserved. The complete decomposition of internal structure and the formation of quartz crystals in this specimen, however, demonstrates that the preservation states of external and internal structures of amber inclusions are not always correlated, as sometimes also seen in Recent specimens.

## AUTHOR CONTRIBUTIONS

**Yan‐Da Li:** Conceptualization (equal); investigation (lead); visualization (equal); writing – original draft (lead); writing – review and editing (lead). **Alfred F. Newton:** Investigation (lead); writing – original draft (equal); writing – review and editing (equal). **Di‐Ying Huang:** Funding acquisition (equal); investigation (equal); writing – review and editing (equal). **Chen‐Yang Cai:** Conceptualization (equal); funding acquisition (equal); investigation (equal); supervision (equal); writing – review and editing (equal).

## CONFLICT OF INTEREST STATEMENT

The authors declare that they have no known competing financial interests or personal relationships that could have appeared to influence the work reported in this paper.

## Data Availability

The original confocal and micro‐CT data are available in the Zenodo repository (https://doi.org/10.5281/zenodo.11200452). This work is registered in ZooBank, under: urn:lsid:zoobank.org:pub:1E7CD956‐888B‐4557‐979D‐6626D1A4286C.
